# HMGB1-modified mesenchymal stem cells attenuate radiation-induced vascular injury possibly via their high motility and facilitation of endothelial differentiation

**DOI:** 10.1186/s13287-019-1197-x

**Published:** 2019-03-13

**Authors:** Xuan Tao, Mingyang Sun, Min Chen, Rongchao Ying, Wenjie Su, Jian Zhang, Xiaodong Xie, Wei Wei, Xiaohu Meng

**Affiliations:** 1 Division of General Surgery, The Second Affiliated Hospital of Nanjing Medical University, Nanjing, China; 2 Department of Gastroenterology, Nanjing University Medical School, Nanjing Drum Tower Hospital, Nanjing, China; 3 Department of Gastroenterological Surgery, Affiliated Hangzhou First People’s Hospital, Zhejiang University School of Medicine, Hangzhou, China; 4 Department of Gastroenterological Surgery, Hangzhou First People’s Hospital, Nanjing Medical University, Hangzhou, China

**Keywords:** Mesenchymal stem cell, Radiation-induced vascular injury, High mobility group box 1, Endothelial cell, CXC chemokine receptor 4, Stromal-derived factor 1

## Abstract

**Background:**

Vascular injury is one of the most common detrimental effects of cancer radiotherapy on healthy tissues. Since the efficacy of current preventive and therapeutic strategies remains limited, the exploration of new approaches to treat radiation-induced vascular injury (RIV) is on high demands. The use of mesenchymal stem cells (MSCs) to treat RIV holds great promise thanks to their well-documented function of mediating tissue regeneration after injury. Recently, we genetically modified MSCs with high mobility group box 1 (HMGB1) and demonstrated the high efficacy of these cells in treating graft atherosclerosis. The current study was to investigate the protective effect of HMGB1-modified MSCs (MSC-H) on RIV by using a rat model.

**Methods:**

Female F344 rats received an intravenous injection of male F344 MSC-H cells or vehicle control at four doses of 2 × 10^6^ cells with a 15-day interval starting from 30 days after irradiation to the abdominal aorta. The aortas were procured for histological and biomedical analysis at 90 days after irradiation. Cell migration to irradiated aortas was traced by green fluorescent protein and sex determination region on the Y chromosome. In vitro cell migration and endothelial differentiation of MSC-H cells were analyzed by stromal-derived factor 1-induced transwell assay and RNA microarray, respectively. The contribution of extracellular HMGB1 to the bioactivity of MSC-H cells was investigated by inhibition experiments with HMGB1 antibody.

**Result:**

MSC-H cell infusion alleviated neointimal formation, vascular inflammation, and fibrosis in irradiated aortas, which was associated with local migration and endothelial differentiation of MSC-H cells. The MSC-H cells showed high motility and potential of endothelial differentiation in vitro. Microarray analysis suggested multiple pathways like MAPK and p53 signaling were activated during endothelial differentiation. MSC-H cells highly expressed CXC chemokine receptor 4 and migrated progressively after stromal-derived factor 1 stimulation, which was blocked by the antagonist of CXC chemokine receptor 4. Finally, the migration and endothelial differentiation of MSC-H cells were inhibited by HMGB1 antibody.

**Conclusion:**

MSC-H cell infusion significantly attenuated RIV, which was associated with their high motility and endothelial differentiation potential. Multiple pathways that possibly contributed to the efficacy of MSC-H cells were suggested and deserved further investigation.

**Electronic supplementary material:**

The online version of this article (10.1186/s13287-019-1197-x) contains supplementary material, which is available to authorized users.

## Background

Radiotherapy remains an important modality of cancer treatment along with surgery and chemotherapy. Approximately one-half of all cancer patients receive radiotherapy to prolong disease-free survival and reduce local recurrence [1]. Besides, radiotherapy contributes to around 40% of curative treatment for cancer [1]. Despite technology-driven improvements in radiotherapy, irradiation of the surrounding healthy tissue takes place in addition to tumor irradiation [2]. Radiotherapy can lead to serious sometimes irreversible injury of normal tissues and organs in the irradiation field, and such injury in some cases is as challenging to treat as the neoplasms themselves [3, 4]. The pathology of radiation-induced injury is varied depending on the affected organs, but the vascular lesions, also referred to as radiation-induced vasculopathy (RIV), are quite consistent, largely relying on the diameter of the vessels [5]. The microvessels are the most radiosensitive and more often subject to acute damage like capillary rupture and thrombosis. In contrast, the injuries to medium-sized and large arteries progress slowly after irradiation but often leading to accelerated atherosclerosis [5–8]. The large arteries become stiffer and less elastic with the breaking and loss of elastic tissues and muscle fibers followed by replacement with extensive fibrosis of media and adventitia, and consequently, diastolic function descends [5, 9]. Moreover, the radiation damage to endothelium gives rise to neointimal proliferation during which the plenty of inflammatory cells and smooth muscle cells gather along the intima to make it thick [9]. The process eventually turns into arterial stenosis, which has been reported to occur in carotid and femoral arteries [10, 11]. Although the aortas have a large luminal diameter and thus rarely develop symptomatic stenosis as carotid and femoral arteries following irradiation, the potentially life-threatening complication of aortic rupture remains a significant clinical concern [5].

Although the precise mechanism of RIV is not fully clear, the dysfunction of endothelial cells (ECs) may play a critical role. Ionizing radiation induces the long-term senescence response in quiescent ECs via generation of oxidative stress and DNA damage [12, 13]. The senescent ECs show a distinct phenotype: the impairment of proliferation and cell-cell junction, inhibition of nitric oxide synthesis, and upregulation of adhesion molecules and proinflammatory cytokines [13–15], which potentially contribute to the pathology of RIV. A group of studies have demonstrated that EC-targeted protection is a potentially effective approach for mitigation of tissue radiotoxicity [16–18]. Nevertheless, the role of irradiated ECs in the current therapeutics for RIV has not been fully appreciated. Thus, new approaches are necessary to replace and reconstitute ECs in the vasculatures that are irreversibly damaged by irradiation. Mesenchymal stem cell (MSC) therapy holds great promise for endothelial regeneration. The review of animal studies and clinical experiences suggests that the efficacy of MSCs lies in both their potential of differentiation toward endothelial lineage and function of secreting various trophic factors to create a nurturing microenvironment for reendothelialization [19]. Moreover, the ability and ease of genetic modification of MSCs have encouraged the discovery of novel molecular targets that will improve the therapeutic effect [20]. Recently, we genetically modified MSCs using ex vivo lentiviral transduction to overexpress high mobility group box 1 (HMGB1) [21]. The HMGB1-modified MSCs (MSC-H) showed high motility and great potential of differentiation toward ECs in vitro and in vivo. Systemically infused MSC-H cells were preferentially recruited to the intima of graft aorta and differentiated to ECs, which was correlated with significant relief of neointimal formation. Inspired by the satisfactory outcomes, we proposed that the delivery of MSC-H cells to the irradiated vessels would yield significant benefits. This study evaluated the hypothesis in a rat model of radiation-induced aortic injury. The possible mechanisms by which HMGB1 modification improved the efficacy of MSCs were also investigated thereafter.

## Materials and methods

### Cell culture and lentiviral transfection

Male F344 rat MSCs (Cyagen Biosciences, China) were adherently cultured in Dulbecco’s modified Eagle’s medium (DMEM) supplemented with 10% fetal bovine serum (Gibco, ThermoFisher Scientific, Waltham, MA, USA). The cells were grown at 37 °C in a humidified atmosphere of 5% CO_2_ and 95% air. The MSCs were transfected with HMGB1-overexpressing vector or vehicle vector to generate MSC-H and MSC-C cells. All lentiviral vectors were constructed by GeneChem (Shanghai, China). The procedures of lentiviral transfection were described previously [21]. Transfection efficiency was assessed by calculating the percentage of green fluorescent protein (GFP)-labeled cells in the general population. The MSC phenotypes and HMGB1 expression were verified by quantitative PCR.

### Animals, study groups, and MSC transplantation

Female F344 rats at 12 weeks of age (with an average body weight of 200 g) were purchased from Vital River Laboratory Animal Technology (Beijing, China). The rats were housed at Laboratory Animal Center of Nanjing Medical University according to “Principles of Laboratory Animal Care” formulated by the National Society for Medical Research and “Guide for the Care and Use of Laboratory Animals” prepared by the Institute of Laboratory Animal Resources and published by National Institutes of Health (NIH Publication No. 86–23, revised 1996). All animal procedures were approved by the Committee of Animal Experiment Ethnics at Nanjing Medical University (Nanjing, China). Totally 32 rats were evenly divided into four groups with eight rats in each group (Additional file 1: Table S1). Aorta irradiation was delivered to all except the rats from Sham RT group. The rats from RT + MSC-H and RT + MSC-C groups received an intravenous injection of MSC-H cells and MSC-C cells, respectively, at the dose of 2 × 10^6^ cells for four times with the interval of 15 days starting from 30 days after irradiation to the abdominal aorta. The MSCs were prepared in serum-free DMEM prior to administration. All the rats were sacrificed 90 days after irradiation, and the abdominal aortas were procured for histological and biomedical analysis.

### Abdominal aorta irradiation

After anesthesia with 10% chloral hydrate, the rat was fixed in supine position. The abdominal skin was prepped by hair shaving followed by disinfection with 70% alcohol. A 5-cm-long midline incision was made to open the abdominal cavity. The small bowel and colon were pulled out of the abdominal cavity, protected by saline-soaked gauze, and left right to the abdomen beyond the field of irradiation. The rat was transferred to the chamber of RS2000 Pro Biological Irradiator (Radsource, USA) and irradiated in a ventrodorsal direction with 160 kV X-ray operating at 25 mA. The total irradiation dosage was 35 Gy which was delivered at the rate of 1.75 Gy/min. The irradiation was localized to a square-shaped field of 3 cm × 3 cm encompassing the abdominal aorta between the infrarenal artery and aortic bifurcation by using a beam-limiting device. The viscera especially small bowel and colon were left off the irradiation field to avoid the devastating gastrointestinal adverse effect. After completion of irradiation, the small bowel and colon were pushed back to the abdominal cavity, and the abdominal incision was closed by 3–0 Vicryl suture. The rat was kept in warming blanket until recovery from anesthesia. For the purpose of study control, the rats undergoing Sham irradiation were given laparotomy without further irradiation.

### Histology analysis

The specimens of aorta were fixed, paraffin-embedded, and cross-sectioned at 5-μm intervals. The sections were stained with hematoxylin-eosin, Masson’s trichrome, and Elastin stain using commercial kits (Servicebio, China). The expression of myeloperoxidase (MPO) in tissue sections was analyzed by using standard avidin-biotin complex technique [22]. The antibodies and reagents were purchased from Agilent Technologies, China, and listed as follows: rabbit polyclonal anti-MPO IgG, biotinylated anti-rabbit IgG, horseradish peroxidase-conjugated streptavidin, and DAB substrate solution. The section images were analyzed by ImageJ software. The area of the vascular lesion (fibrosis, inflammation, and MPO staining) was normalized to the total area of the vascular wall and expressed in percentage. The thickness of intima was divided by the full thickness of the vascular wall and expressed in percentage.

### Cell and tissue immunofluorescence

The sections of fresh aortas and glass coverslips containing cultured cells were fixed with cold acetone and blocked by 10% goat serum. CD31 was probed with mouse anti-CD31 IgG and then visualized by incubation with Alexa Fluor 647 conjugated goat anti-mouse IgG antibodies (Abcam, Shanghai, China). The nuclei were stained with 4′,6-diamidino-2-phenylindole (DAPI).

### Low-density lipoprotein (LDL) uptake assay

After incubation in serum-free DMEM overnight, the cells were treated with 5 μg/ml DiI-Ac-LDL (ThermoFisher Scientific, MA) for 4 h. The nuclei were counter-stained with DAPI for 10 min. The cell uptake of LDL was visualized by fluorescent microscopy.

### Quantitative PCR

For PCR analysis, a total of 40 mg aortic tissue was collected from eight rats of the same group with 5 mg from each rat. The cell sample was prepared from 1 × 10^6^ cells. Total RNA samples were extracted from the tissue and cells by using TRIzol reagent (Invitrogen, USA) according to the manufacturer’s protocols. The RNA purity was assessed by spectrometry, and the quality and integrity of the total RNA were investigated by Agilent Bioanalyzer 2100. The reverse transcription was conducted by using the 5 × PrimeScript RT Master Mix (Takara, Dalian, China) to synthesize first-strand complementary DNA from the total RNA samples. Then the real-time quantitative PCR to determine the relative RNA levels was performed on Applied Biosystem 7500 by using the SYBR Green method (Takara, Dalian, China). The primers were prepared by Sangon Biotech, China according to the reported sequence [23–25]: tumor necrosis factor α (TNF-α) forward primer 5′-CACGCTCTTCTGTCTACTGA-3′ and reverse primer 5′-GGACTCCGTGATGTCTAAGT-3′, transforming growth factor β (TGF-β) forward primer 5′-CCTGGGCACCATCCATGA-3′ and reverse primer 5′-CAGGTGTTGAGCCCTTTCCA-3′, interleukin-1β (IL-1β) forward primer 5′-GGGTTGAATCTATACCTGTCCTGTGT-3′ and reverse primer 5′-GACAAACCGCTTTTCCATCTTCT-3′, stromal cell-derived factor-1 (SDF-1) forward primer: GAAGAGAAGCCATAGTAGT and reverse primer: GCATATAGTGTCACAGTTG, CXC chemokine receptor 4 (CXCR4) forward primer: 5′-ACTGCATCATCATCTCCAAGC-3′ and reverse primer: 5′-CTCTCGAAGTCACATCCTTGC-3′, Glyceraldehyde-3-phosphate dehydrogenase (GAPDH) forward primer: 5′-GGGGCTCTCTGCTCCTCCCTGT-3′ and reverse primer: 5′-CGGCCAAATCCGTTCACACCGA-3′. GAPDH served as an internal control, and the fold change in gene expression was calculated using 2^−ΔΔCT^ method.

The sex determination region on the Y chromosome (Sry) gene in the aortic tissue was quantified by real-time quantitative PCR. First, total DNA was extracted by using a genomic DNA minipreparation kit with a spin column (Beyotime, China). The PCR was performed on Roche LightCycler system by using 2 × SYBR Green qPCR Master Mix (Takara, China). The primers for Sry and β-actin genes were synthesized by Sangon Biotech, China, according to the reported sequence [26]: Sry forward primer 5′-GAGGCACAAGTTGGCTCAACA-3′ and reverse primer 5′-CTCCTGCAAAAAGGGCCTTT-3′, β-actin forward primer 5′-CCATTGAACACGGCATTG-3′ and reverse primer 5′-TACGACCAGAGGCATACA-3′. The Sry DNA level was normalized to that of β-actin by using 2^−ΔΔCT^ method.

### Microarray analysis of gene expression profile

Microarray analysis of MSC gene expression profile was performed by GeneChem (Shanghai, China). Gene expression was investigated with rat Clariom S arrays (ThermoFisher Scientific, MA, USA) by using total RNA for reverse transcription. The reverse transcription was initiated at the poly-A tail and throughout the RNA length. The complementary RNA amplification was obtained by low-cycle PCR followed by linear amplification through T7 in vitro transcription. After purification and quantification by a NanoDrop spectrophotometer, the complementary RNA was converted into biotinylated single-stranded complementary DNA. After purification and quantification by a NanoDrop spectrophotometer, the complementary DNA was fragmented and terminally labeled for hybridization to arrays. The hybridized arrays were then stained, washed, and scanned using a GeneChip Fluidics Station 450, Command Console Software, and GeneChip Scanner 3000 7G. Images were scanned by Affymetrix GeneChip Command Console and analyzed with the Affymetrix GeneChip Expression Console. Data quality was estimated by confirming the order of signal intensities of the poly-A and hybridization controls with Expression Console Software. Molecular function and signaling pathways in which differentially expressed genes were enriched were analyzed by using Gene Ontology (GO) and Kyoto Encyclopedia of Genes and Genomes (KEGG), respectively.

### EC-MSC coculture system

The EC-MSC coculture system was reported previously [27]. Each well of 6-well plates was separated to the upper and lower chamber by a Transwell insert with the 0.4-μm-pore membrane. The lower chamber was loaded with the suspension of 3 × 10^4^ MSCs, and 1 × 10^5^ mouse microvascular ECs (Creative Bioarray, Shirley, NY) were seeded in the upper chamber. To test the effect of HMGB1 autocrine signaling, HMGB1 antibody (HMGB1 Ab) from Abcam at 1:1000 dilution was added to the lower chamber. The indirect cocultures were maintained in EGM-2 medium (Lonza, Walkersville, MD) containing 1% fetal bovine serum (Gibco, ThermoFisher Scientific, Waltham, MA, USA) for 14 days. During the period, the medium was changed every 3 to 4 days, and an aliquot of MSCs was taken from the cocultures for PCR analysis of CD31 expression. At day 14, the cells with endothelial differentiation were identified by CD31 immunostaining and LDL uptake assay. The experiments were repeated three times for each group.

### Transwell migration assay

The cell migration was evaluated by transwell assay. The assay was performed in Corning 24-well plates. The upper and lower compartments were separated by a chamber insert with 8.0-μm-pore filter membrane at the bottom. The upper compartment was loaded with 1 × 10^5^ cells and serum-free DMEM, and the lower compartment contained DMEM supplemented with 15% fetal bovine serum. To test the effect of HMGB1 autocrine signaling, HMGB1 Ab (1:1000 dilution) was added to the upper compartment. After being cultured for 24 h, the cells that migrated to the lower side of the membrane were fixed in methanol, stained with 0.1% crystal violet solution, and visualized by phase-contrast microscopy. To evaluate the chemotactic effect of SDF-1 on cell migration, the upper compartment was mounted with 1 × 10^5^ cells and DMEM. The lower compartment was loaded with DMEM containing SDF-1 (PeproTech, NJ) at a concentration gradient of 0, 25, 50, 100, and 200 ng/ml. After incubation for 6 h at 37 °C and 5% CO_2_, the cells that migrated through the filters were fixed with methanol and stained with 0.5% crystal violet. For SDF-1/CXCR4 inhibition experiments, MSCs were pre-incubated for 30 min with 5 μg/ml AMD3100, a specific CXCR4 antagonist [25]. The experiments were repeated five times for each group, and data were expressed as the average migrated cell count per × 200 field micrograph.

### Fractionation of cytosolic and nuclear proteins

The cytosolic and nuclear fractions from cultured cells were obtained as previously described [28]. Briefly, cell samples were incubated on ice for 10 min in cytosolic lysis buffer (10 mM Tris-HCl pH 8.0, 60 mM KCl, 1 mM EDTA, protease inhibitor and 0.5% NP-40) and gently triturated using a 26-gauge needle. After centrifugation at 3000 rpm for 5 min at 4 °C, the supernatant was collected as the cytosolic fraction. The pellet was re-suspended in nuclear lysis buffer (20 mM Tris-HCl, pH 8.0, 420 mM NaCl, 0.2 mM EDTA, 25% glycerol, 1.5 mM MgCl_2_, protease inhibitor, 1% Triton-X100 in ultrapure water) supplemented with DNase (Beyotime, China), incubated on ice for 30 min, repeatedly triturated, and centrifuged at 14,000 rpm for 10 min at 4 °C. The resulting supernatant was retained as the nuclear fraction.

### Western blot analysis

The whole cell lysates were prepared by using RIPA lysis buffer containing PMSF (Beyotime, China). The cytosolic and nuclear fractions were prepared as aforementioned. Protein samples were separated using 12% SDS-PAGE gel, transferred to polyvinylidene difluoride membranes, and blocked with 5% nonfat milk solution. The membranes were incubated with primary antibody overnight followed by the corresponding secondary antibody for 1 h. Antibodies were obtained from Abcam (Shanghai, China) and listed as follows: rabbit anti-HMGB1 IgG, rabbit anti-Lamin B1 IgG, rabbit anti-CXCR4 IgG, rabbit anti-tubulin IgG, and horseradish peroxidase-conjugated goat anti-rabbit IgG. Tubulin served as the internal control for whole cell lysates and cytosolic fractions. Lamin B1 served as the internal control for nuclear fractions. Antibody binding was visualized using the BeyoECL Star system (Beyotime, China).

### Extracellular HMGB1 measurement

Cells were cultured in 12-well plates. Extracellular HMGB1 in cell cultures was measured by Western blot analysis as previously described [29]. Briefly, equal volumes of cell culture supernatant were obtained from each well and subjected to 12% SDS-PAGE electrophoresis. The resolved protein was transferred to PVDF membrane. After being blocked with 5% nonfat milk solution, the membranes were incubated with rabbit anti-HMGB1 IgG overnight followed by horseradish peroxidase-conjugated goat anti-rabbit secondary antibody for 1 h. HMGB1 band was visualized with the ECL reagents. The band intensity was measured by ImageJ software. By using the same protocol, a series of standard samples with known quantities of recombinant HMGB1 (Sigma-Aldrich, USA) were analyzed to generate a standard curve. By comparing the band intensity with the standard curve, the assay determined how much HMGB1 was released by cultured cells to the supernatant. Values were normalized to the total cell number in each well and expressed in nanograms per 10^6^ cells. To control for the differences among wells in cell survival, the activity of lactate dehydrogenase (LDH) in each well was quantified by using TOX-7 kit from Sigma. The LDH activity in the culture supernatant was measured at first. Then the cells were lysed by repeated cycles of freezing and thawing, and LDH activity in each well was measured again to obtain the activity of total LDH. The LDH activity in the supernatant was normalized to the activity of total LDH in each well and expressed in percentage. The percentage was the relative amount of LDH release and positively correlated with the cell death rate.

### Statistical analysis

All experiments were repeated for at least three times. Data were processed using GraphPad Prism 5 (GraphPad Software, Saint Diego, CA, USA). Data are expressed as mean ± standard deviation and compared between groups by the Mann-Whitney test or Student *t* test. A *P* value of < 0.05 was considered statistically significant.

## Results

### MSC-H cell infusion alleviated neointimal formation, vascular inflammation, and fibrosis in irradiated aortas

**Fig. 1:**
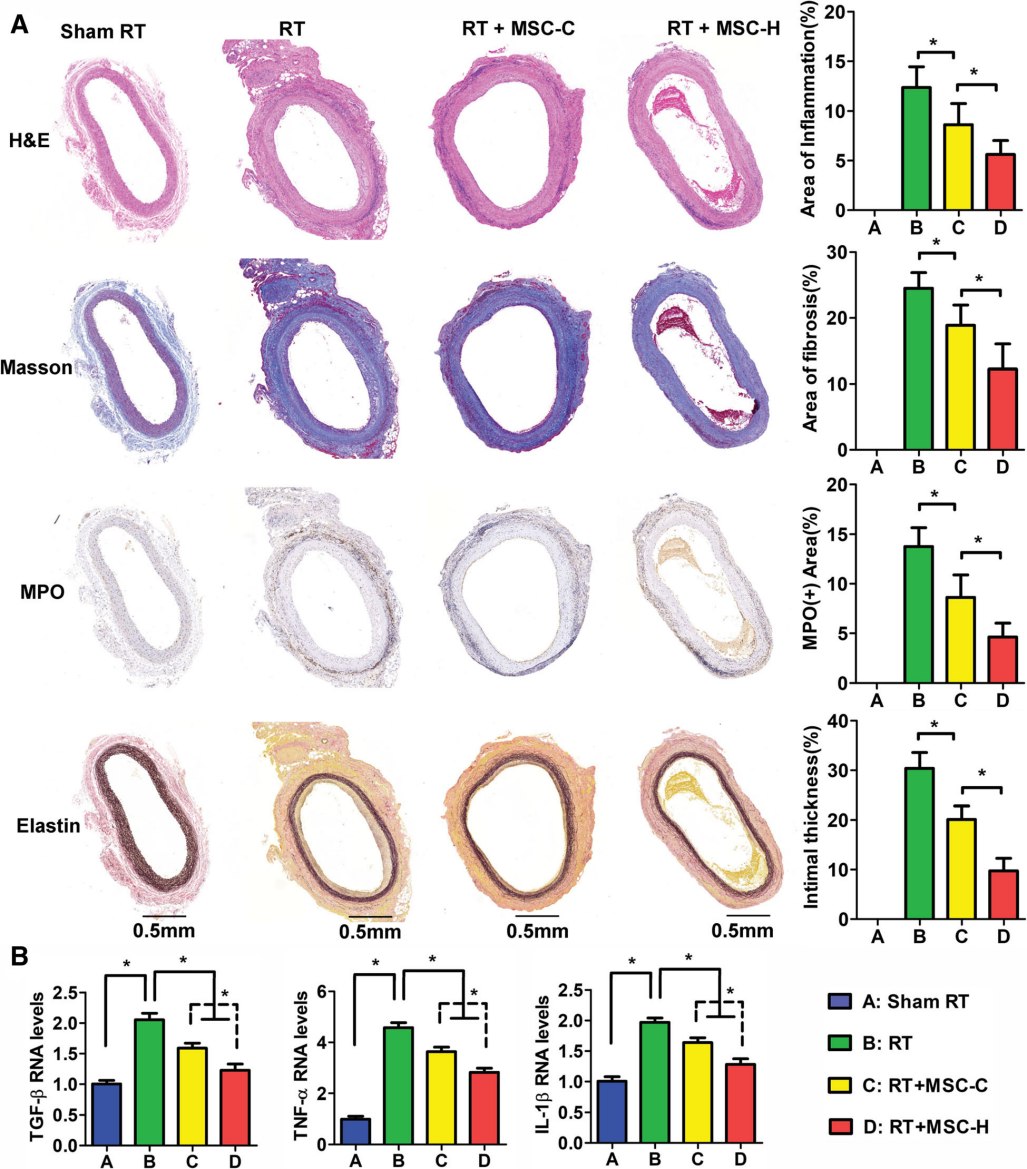
Histology and biomedical analysis of the aortas at day 90 after irradiation. **a** The sections of the aortas were stained hematoxylin-eosin (H&E), Masson’s trichrome, and Elastin stain and immunostained against myeloperoxidase (MPO). The area of the vascular lesion (fibrosis, inflammation, and MPO staining) was normalized to the total area of the vascular wall and expressed in percentage. The thickness of intima was divided by the full thickness of the vascular wall and expressed in percentage. The irradiated aortas (RT, RT + MSC-C, and RT + MSC-H groups) showed extensive inflammation, diffuse fibrosis, and neointimal formation. In H&E stain, the plenty of inflammatory cells infiltrated to the arterial adventitia. These cells were positive for MPO staining. The elastic fibers that normally appeared as brown and waved lines after elastin staining were decreased in the media and replaced by bright blue collagen fibers in Masson’s trichrome stain. The collagen fibers not only existed in the media layer but spread across the entire arterial wall. The neointima was clearly visible in H&E and Elastin staining. Despite the absence of injury in Sham RT group, the degree of vascular injury was varied among groups with irradiation. The area of inflammation, fibrosis, and MPO staining as well as intimal thickness was greatest in the RT group, less in the RT + MSC-C group, and lowest in the RT + MSC-H group. Eight rats were examined for each group, and the representative section images were shown. Group comparisons were made using the Mann-Whitney test. *P < 0.05. **b** The RNA levels of TNF-α, TGF-β, and IL-1β in the aortas were measured by quantitative PCR. The assay was repeated three times for each sample, and the RNA level was expressed in the fold change to that of Sham RT group. Group comparisons were made by using Student’s t test. *P < 0.05

Ninety days after aorta irradiation, the segment of affected aortas was procured for histological analysis. The irradiated aortas showed extensive inflammation, diffuse fibrosis, and neointimal formation which were in accordance with the reported vascular injury after irradiation in humans [5] (Fig. 1a, RT group). The neointima was formed by the gathering of abundant spindle-like cells and extracellular matrix mixed with some degree of inflammatory cell infiltration internal to the elastic membrane. The elastic fibers that normally appeared as brown and waved lines after elastin staining were decreased in the media and replaced by bright blue collagen fibers in Masson’s trichrome stain. The collagen fibers not only existed in the media layer but spread across the entire arterial wall, suggesting diffuse fibrosis of irradiated aortas. Moreover, the irradiated aortas showed strong oxidative stress response as the plenty of MPO-positive inflammatory cells accumulated in arterial adventitia after irradiation. In contrast, all the histological changes were absent in Sham RT group whose aortas remained almost normal (Fig. 1a, Sham RT group). MSC infusion significantly reduced neointimal formation, vascular fibrosis, and inflammatory cell infiltration (Fig. 1a, RT + MSC-C and RT + MSC-H groups). Moreover, the rats with MSC-H treatment (RT + MSC-H group) demonstrated significant histological relief of vascular injury when compared with those receiving MSC-C treatment (RT + MSC-C group). Among the groups with irradiation, the area of inflammation, fibrosis, and MPO staining as well as intimal thickness was greatest in RT group, less in RT + MSC-C group, and lowest in RT + MSC-H group. To further assess the inflammatory response, we analyzed the levels of TNF-α, TGF-β, and IL-1 in the homogenate of irradiated aortas. All the inflammatory cytokines were remarkably increased in irradiated aortas, suggesting a profound inflammatory response to irradiation. However, MSC infusion significantly reduced the cytokine levels, and notably, a more remarkable decline of these cytokine levels was yielded by the RT + MSC-H group (Fig. 1b).

### MSC-H cells showed high motility and potential of endothelial differentiation in vivo and in vitro

**Fig. 2:**
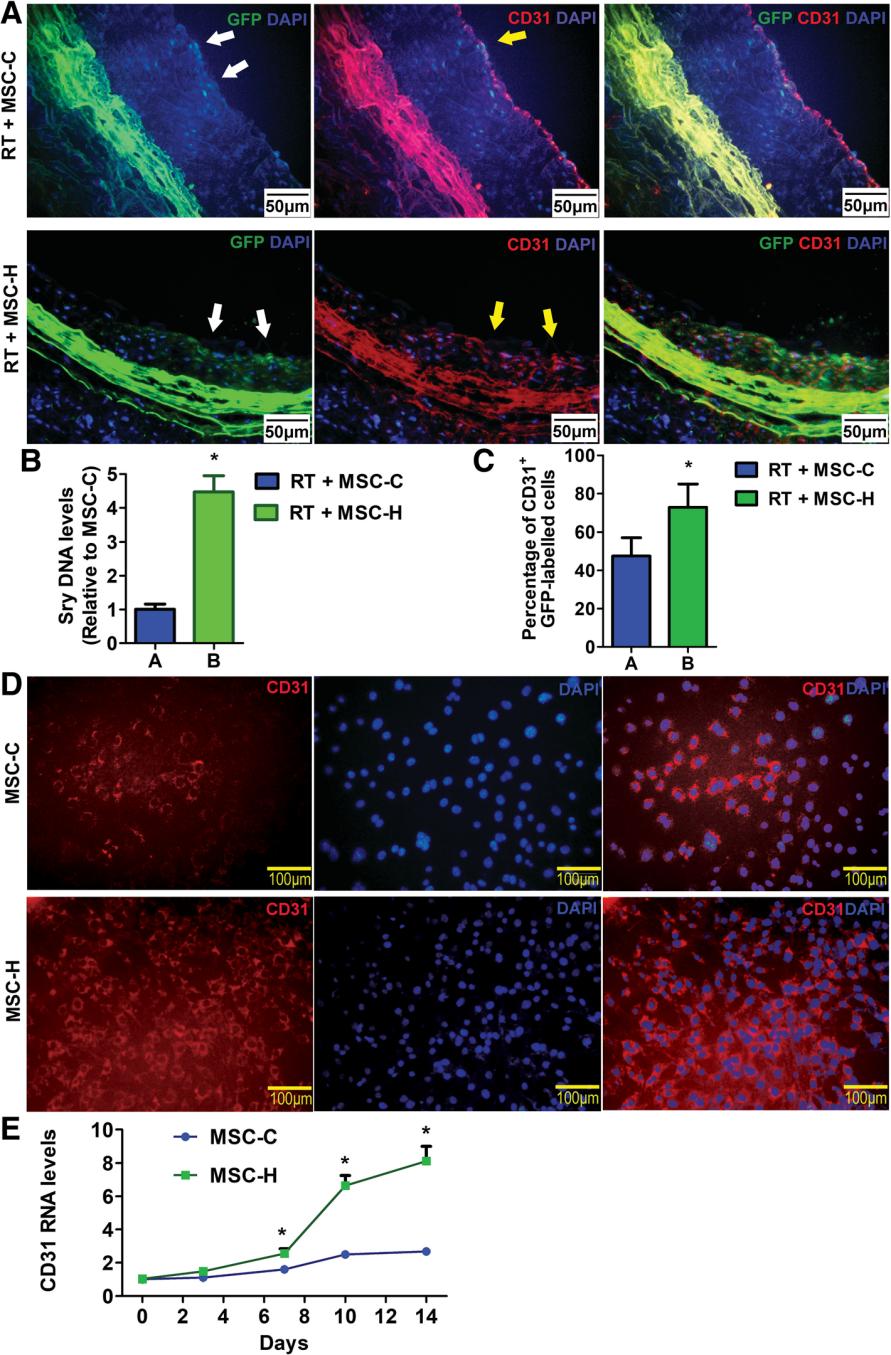
The migration and endothelial differentiation of MSCs in vivo and in vitro. **a** Local cell migration to irradiated aortas was traced by GFP label. The GFP-labeled MSCs were visible in the neointima, suggesting cell implantation in the irradiated aortas. RT + MSC-H group had a larger population of migrated cells than RT + MSC-C group. The ECs in the neointima were identified by CD31. The majority of migrated cells from MSC-H group expressed CD31. **b** The levels of Sry gene in the aortas were measured by quantitative PCR. RT + MSC-H group yield a higher level of Sry gene than the RT + MSC-C group. The assay was repeated three times for each sample, and the RNA level was expressed in the fold change to that of RT + MSC-C group. Group comparison was made by using Student’s *t* test. *P < 0.05. **c** The percentage of CD31-positive cells in migrated MSCs were calculated and compared between groups. RT + MSC-H group had a higher percentage of migrated cells expressing CD31 than RT + MSC-C group. Five random high-power fields were examined in the sections of the aorta for each rat, and CD31-positive GFP-labeled cells were counted to determine the percentage of migrated cells expressing CD31. The average percentage was calculated from eight rats for each group. Group comparison was made using the Mann-Whitney test. *P < 0.05. **d** In vitro endothelial differentiation of MSCs was generated by EC-MSC coculture system. The ECs were identified by CD31. A greater number of MSC-H cells differentiated to ECs than MSC-C cells on day 14. **e** The RNA levels of CD31 in MSCs were measured by quantitative PCR during the coculture. A gradual increase of CD31 RNA levels was acquired by both MSC-C and MSC-H cells from day 7 till day 14. In comparison, MSC-H cells yielded higher levels of CD31 RNA than MSC-C cells since day 7. The assay was repeated three times for each sample, and the RNA level was expressed in the fold change to that of day 0. Group comparisons were made by using Student’s *t* test. *P < 0.05

By using a rat model of graft atherosclerosis, we found that MSC-H cells had a favorable effect on reducing neointima formation, partly attributable to their high motility and potential of differentiation toward endothelial lineage. These findings were further validated in the RIV model. The MSCs carried GFP and Sry genes so that their local migration was traced after systemic administration. Their endothelial differentiation was identified by CD31. Consequently, when compared to the negative control groups (Additional file 2: Figure S1), the migrated MSCs were visible in RT+MSC-C and RT+ MSC-H groups. A significantly larger population of MSC-H cells was found at irradiated aortas especially gathering along the neointima when compared to the MSC-C cells (Fig. 2a). RT + MSC-H group had a significantly higher level of Sry gene than the RT + MSC-C group (Fig. 2b), suggesting more efficient implantation of MSC-H cells to the irradiated aortas than MSC-C cells. Moreover, the proportion of CD31-positive cells to total GFP-labeled cells was significantly higher in RT + MSC-H group than RT + MSC-C group, suggesting MSC-H cells preferentially differentiated into ECs in vivo (Fig. 2c). This was correlated with the inhibition of neointimal formation in the RT + MSC-H group. Next, we verified in vitro endothelial differentiation capacity of MSC-H cells by using EC-MSC coculture technique. Unlike VEGF-induced endothelial differentiation [21], this technique is appropriate to use in the scenario where MSCs grow and differentiate as naturally as at angiogenic microenvironment in vivo [30]. The expression of CD31 and cell uptake of LDL was used to determine endothelial differentiation during the coculture. As a result, a significantly larger population of MSC-H cells had CD31-positive staining and LDL uptake than MSC-C cells at day 14 (Fig. 2d, Additional file 3: Figure S2). During the coculture, a gradual increase of CD31 RNA was acquired by both MSC-C and MSC-H cells from day 7 to day 14. In comparison, MSC-H cells yielded a higher level of CD31 RNA than MSC-C cells since day 7 (Fig. 2e). Taken together, the present study agreed with our previous findings and showed high motility and EC differentiation potential of MSC-H cells in vivo and in vitro.

### Microarray analysis of gene expression profiles in MSC-H cells during endothelial differentiation

**Fig. 3:**
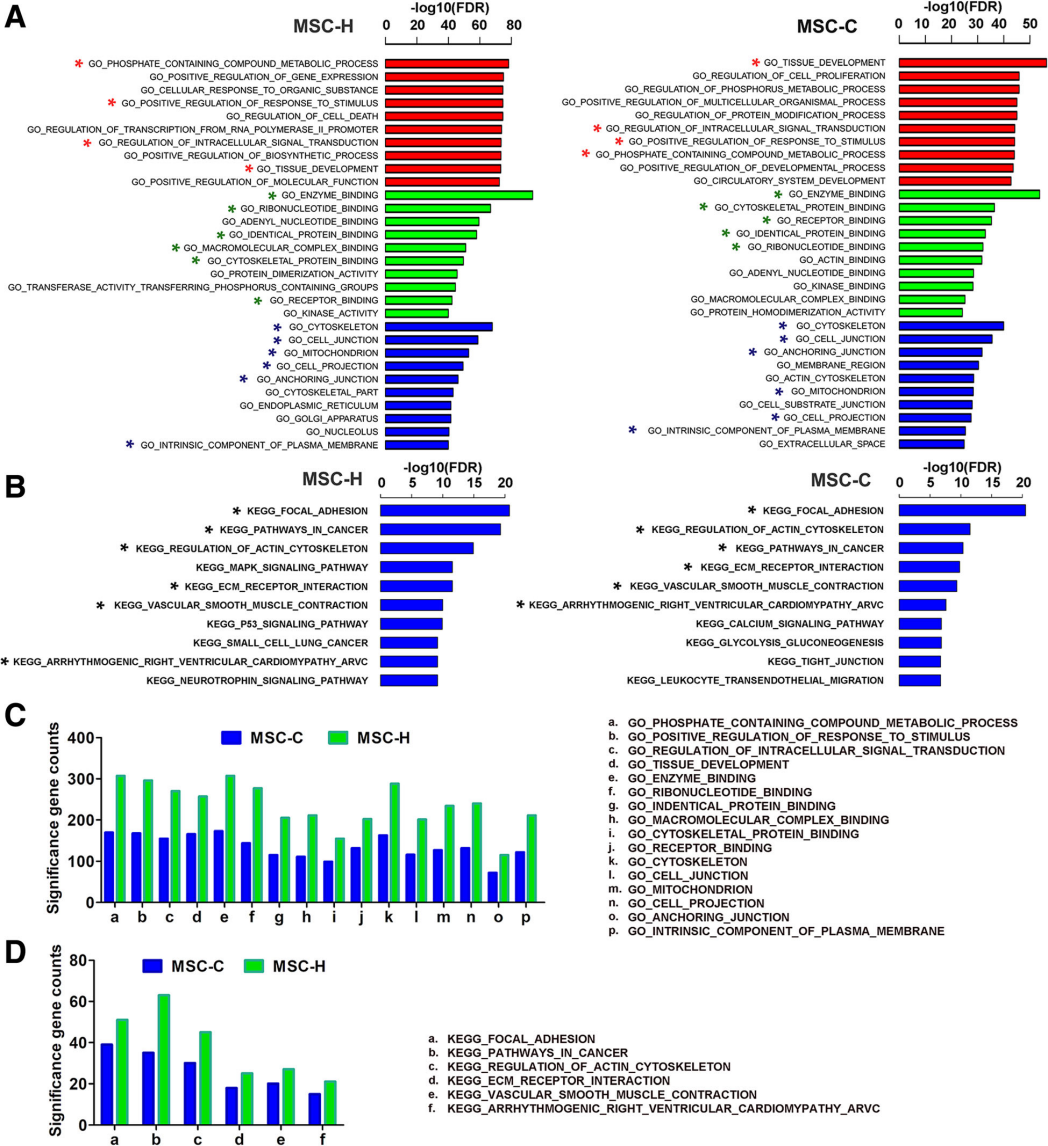
Microarray analysis of differentially expressed genes in MSCs during endothelial differentiation. In EC-MSC coculture system, there MSC samples for each group were collected at days 0 and 7, respectively, and sent to microarray analysis of differentially expressed genes. The inclusion criteria for differentially expressed genes were the absolute fold change of RNA levels > 1.5 and *P* value < 0.05. The analysis yielded 2482 (999 upregulated and 1483 downregulated) genes in MSC-H cells and 1325 (671 upregulated and 654 downregulated) genes in MSC-C cells. These genes were enriched in GO terms (**a**) and KEGG pathways analysis (**b**). The enriched terms that were present in both MSC-C and MSC-H groups were marked by asterisks. The MSC-H cells yielded a cluster of the most enriched terms with high similarity to MSC-C cells. Approximately one-half of the most enriched GO terms and pathways were consistent between them. Nevertheless, multiple pathways were noteworthy of being exclusively enriched in MSC-H cells, including MAPK and p53 signaling. **c** The number of differentially expressed genes (significance genes) enriched in the shared GO terms was counted and compared between groups. MSC-H cells yielded 1.7-fold (ranged from 1.5 to 1.9-fold) greater number of significance genes than MSC-C cells on average. **d** The number of differentially expressed genes (significance genes) enriched in the shared signaling pathways was counted and compared between groups. Similarly, MSC-H cells had an average 1.4-fold (ranged from 1.3 to 1.8-fold) increase of significance gene counts than the MSC-H cells

To further clarify how HMGB1 overexpression promoted MSC differentiation toward endothelial lineage, we performed microarray analysis of differentially expressed genes in MSC-H cells and MSC-C cells after coculture with ECs for 7 days. The inclusion criteria for differentially expressed genes were the absolute fold change of RNA levels > 1.5 and *P* value < 0.05. The analysis yielded 2482 (999 upregulated and 1483 downregulated) genes in MSC-H cells and 1325 (671 upregulated and 654 downregulated) genes in MSC-C cells. MSC-H cells had a larger subset of differentially expressed genes (nearly two-fold of significance gene counts) than MSC-C cells. These genes were processed with GO enrichment and KEGG pathway analysis. As a result, MSC-H cells showed the gene enrichment patterns similar to MSC-C cells in that approximately one-half of the most enriched GO terms and pathways were consistent between them (Fig. 3a, b). Among the shared GO terms, the significance gene counts of MSC-H cells, however, were 1.7-fold (ranged from 1.5 to 1.9-fold) higher on average than those of MSC-C cells (Fig. 3c). Similarly, MSC-H cells yielded an average 1.4-fold (ranged from 1.3 to 1.8-fold) increase of significance gene counts than MSC-H cells among the shared pathways (Fig. 3d). Nevertheless, a cluster of pathways were exclusively enriched in MSC-H cells rather than MSC-H cells, including MAPK and p53 signaling pathways (Fig. 3b) (Additional file 4).

### The motility of MSC-H cells was regulated by the SDF-1/CXCR4 axis

**Fig. 4:**
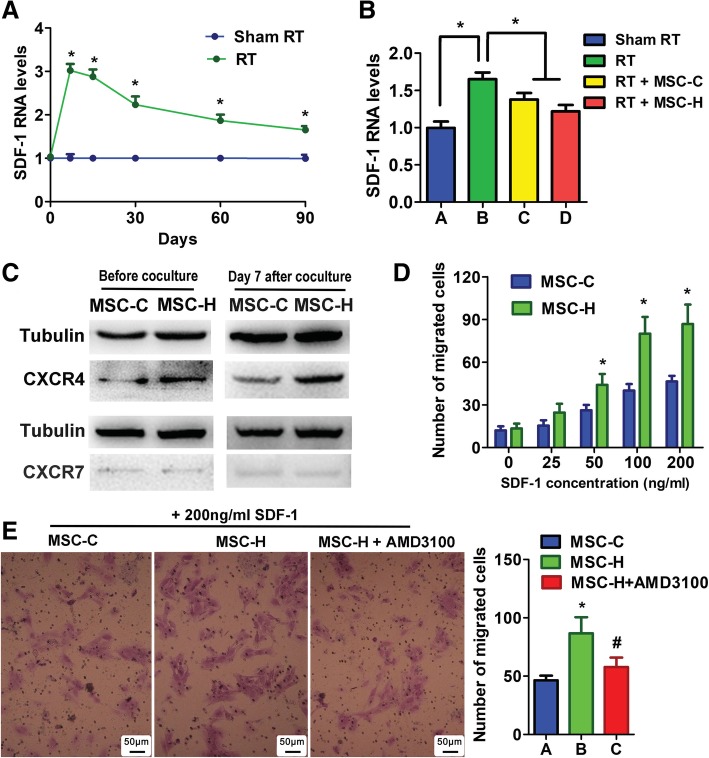
The association of SDF-1/CXCR4 axis with high motility of MSC-H cells. **a** The SDF-1 expression in the aortic specimens were measured by quantitative PCR. The assay was repeated three times for each group, and the RNA level was expressed in the fold change to the level at day 0. The SDF-1 expression was increased by three-fold in the irradiated aortas (RT group) on day 7 after irradiation and then dropped down gradually to the 1.6-fold increase level at day 90. In comparison, the expression of SDF-1 in nonirradiated aortas (Sham RT group) kept stable throughout the experiment. An asterisk indicated that the SDF-1 levels were statistically different *(P* < 0.05) between RT and Sham RT group at a specific time checkpoint. **b** The SDF-1 expression was compared between groups with and without MSC treatment at day 90 after irradiation. The SDF-1 level was significantly lower in MSC-treated groups (RT + MSC-C and RT + MSC-H). Although RT + MSC-H group yielded a lower average SDF-1 level than RT + MSC-C group, the difference did not reach statistical significance. The assay was repeated three times for each group, and the RNA level was expressed in the fold change to that of Sham RT group. Group comparisons were made by using Student’s *t* test. *P < 0.05. **c** The expression of CXCR4 and CXCR7 in MSCs were measured by Western blot. The assay was repeated three times for each cell type, and the representative images of protein bands were shown. The CXCR4 expression was significantly higher in MSC-H cells than MSC-C cells. However, both of them expressed a low level of CXCR7. The similar results were obtained after MSC-H and MSC-C cells were cocultured with ECs for 7 days. **d** The mobility of MSCs was assessed by SDF-1-induced transwell migration assay and expressed as a number of migrated cells per high powered field. The cell migration was analyzed after stimulation with SDF-1 at the concentration gradient of 25, 50, 100, and 200 ng/ml. The number of migrated cells was significantly higher in MSC-H cells than MSC-C cells when the SDF-1 concentration increased to 50 ng/ml or more. The assays were repeated five times for each group. Group comparisons were made by using Student’s *t* test. *P < 0.05. **e** The MSC-H cell migration stimulated by 200 ng/ml SDF-1 was inhibited by the CXCR4 antagonist, AMD3100. The assays were repeated five times for each group. Group comparisons were made by using Student’s *t* test. *P < 0.05 MSC-H vs MSC-C. # *P* < 0.05 MSC-H + AMD3100 vs MSC-H

To investigate whether the SDF1 signaling pathway was associated with high motility of MSC-H cells, we measured the SDF-1 level in the irradiated aortas. In the preliminary experiment, the expression of SDF-1 in the aortas was measured at 7, 15, 30, 60, and 90 days after irradiation. The SDF-1 level of irradiated aortas (RT group) dramatically rose to the maximum of three-fold increase at day 7 and then dropped down gradually to the 1.6-fold increase level at day 90. In comparison, the SDF-1 levels were unaltered over time in the aortas without irradiation (Sham RT group). RT group had significantly higher levels of SDF-1 than Sham RT group during the 90 days (Fig. 4a). After MSC infusion, the SDF-1 levels were reduced, which was in accordance with the decline of other inflammatory cytokines that were measured in the irradiated aortas (Figs. 4b and 1b). Then we investigated the expression of two SDF-1 receptors, CXCR4 and CXCR7 (CXC chemokine receptor 7), on MSC-H cells. The expression of CXCR4 was significantly higher in MSC-H cells than MSC-C cells while both of them expressed a very low level of CXCR7. The similar results were obtained on day 7 after EC-MSC coculture (Fig. 4c). These findings suggested that the SDF-1/CXCR4 axis rather than SDF-1/CXCR7 axis was linked to the enhanced migration of MSC-H cells. To support their causal relationship, we conducted SDF-1-induced cell migration assay. SDF-1 stimulated MSC-H and MSC-C cells to migrate in a concentrate-dependent manner (Fig. 4d). But the number of migrated MSC-H cells was significantly greater than MSC-C cells when SDF-1 concentration increased to 50 ng/ml and higher, suggesting the enhanced response of MSC-H cells to SDF-1 stimulation. After MSC-H cells were treated with AMD3100, the specific antagonist of CXCR4, the cell migration was significantly inhibited (Fig. 4e). Taken together, the study revealed that the motility of MSC-H cells was at least partly regulated by the SDF-1/CXCR4 axis.

### MSC-H cells were activated via HMGB1 autocrine signaling

**Fig. 5:**
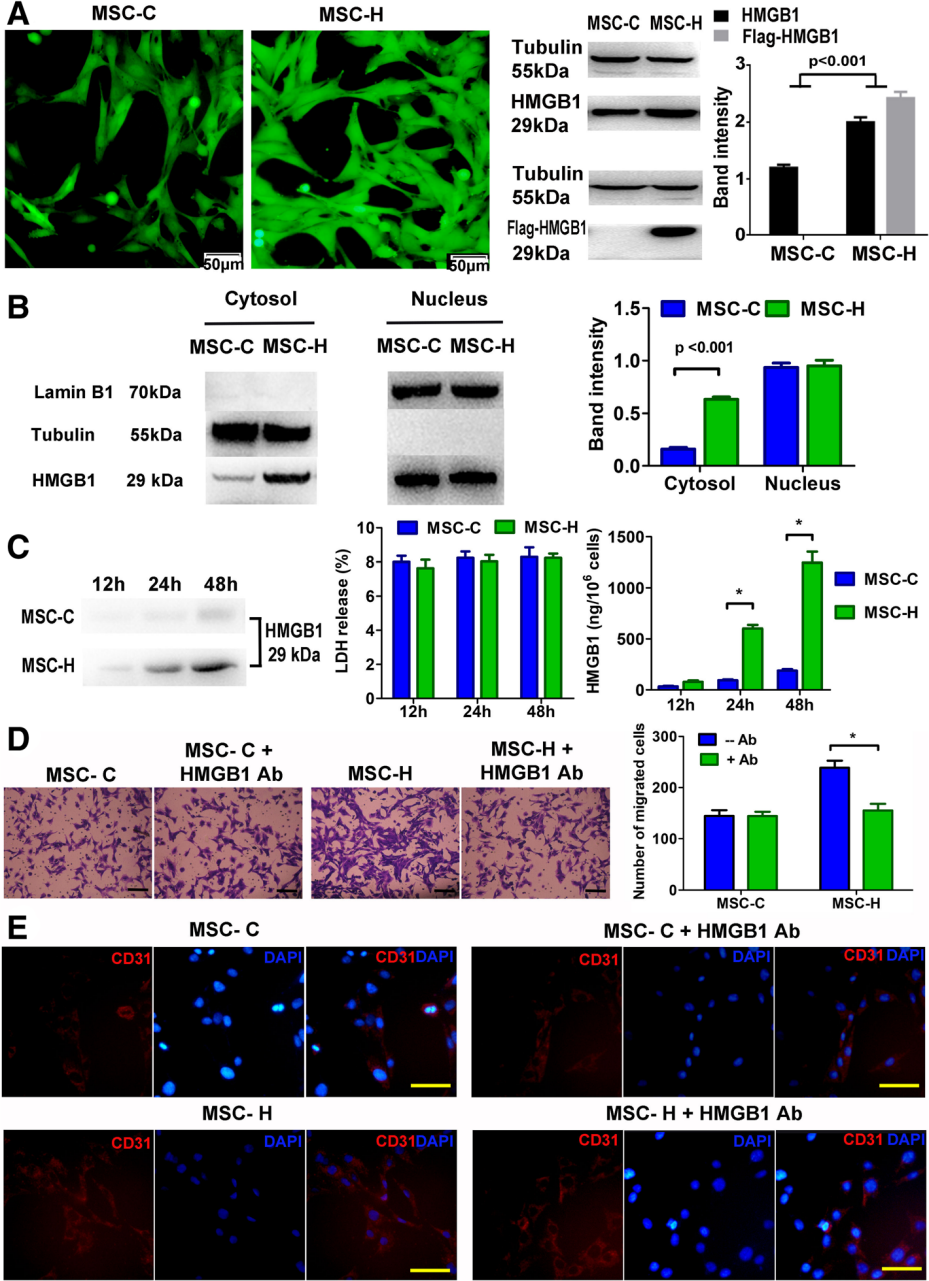
MSC-H cells were activated by HMGB1 autocrine signaling. **a** Transfection of MSCs with HMGB1 overexpressing vectors and vehicle control vectors yielded MSC-H and MSC-C cells, respectively. Both of them expressed GFP, rendering them visible under a fluorescent microscope. The level of HMGB1 was 1.5-fold higher in the whole cell lysate from MSC-H cells than MSC-C cells. The MSC-H cells expressed HMGB1 with flag tag which was generated by viral transfection. The data shown were representative of three repeated tests for each group. **b** The level of cytosolic HMGB1 was nearly four-fold higher in MSC-H cells than MSC-C cells. But the HMGB1 levels in the nucleus were almost consistent between them. Tubulin and Lamin B1 served as the quality control for cytosolic and nuclear fractionation. The data shown were representative of three repeated tests for each group. **c** The production of extracellular HMGB1 was at low levels for both MSC-H and MSC-C cells after culture for 12 h. However, MSC-H cells generated a greater amount of extracellular HMGB1, approximately six-fold higher than MSC-C cells after culture for 24 h and 48 h. The difference of LDH release among the groups was not statistically significant, suggesting the increase of extracellular HMGB1 was not due to cell death. The assays were repeated three times for each group. Group comparisons were made by using Student’s *t* test. *P < 0.05. **d** The number of migrated MSC-H cells was significantly reduced by HMGB1 antibody (HMGB1 Ab). The assays were repeated three times for each group. Group comparisons were made by using Student’s *t* test. *P < 0.05. Scale bar = 50 μm. **e** In the EC-MSC coculture system, the population of CD31-positive cells was significantly decreased at day 14 after the MSC-H cells were treated with HMGB1 Ab. The images were representative of three repeated tests for each group. Scale bar = 50 μm

To explore whether MSC-H cells were activated via HMGB1 autocrine signaling, we analyzed HMGB1 production and release in MSC-H cells. Although the HMGB1 level in whole cell lysate was increased slightly by 1.5-fold after transfection with HMGB1 overexpressing vectors (Fig. 5a), the level of cytosolic HMGB1 was four-fold higher in MSC-H cells than MSC-C cells (Fig. 5b). In comparison, the HMGB1 levels in the nucleus were not different between MSC-H and MSC-C cells (Fig. 5b). Then the HMGB1 concentration in the culture supernatant was measured and calculated to the amount of extracellular HMGB1 released from the cultured cells. MSC-H and MSC-C cells generated a low amount of extracellular HMGB1 after culture for 12 h. However, the production of extracellular HMGB1 by MSC-H cells was dramatically increased to six-fold higher than the amount of HMGB1 produced by MSC-C cells after culture for 24 h and 48 h. The LDH release from MSC-H cells and MSC-C cells was not significantly different (Fig. 5c). To further validate the role of extracellular HMGB1 in the bioactivity of MSC-H cells, we investigated whether neutralization of extracellular HMGB1 by HMGB1 Ab would inhibit the migration and endothelial differentiation of MSC-H cells. The migration of MSC-H cells was significantly inhibited by HMGB1 Ab (Fig. 5d). The population of MSC-H cells differentiating to CD31-positive cells was reduced by HMGB1 Ab (Fig. 5e), which was in accordance with the impairment of LDL uptake (Additional file 3: Figure S2). Taken together, these findings suggested that HMGB1 autocrine signaling was essential to the bioactivity of MSC-H cells.

## Discussion

The clinical use of radiotherapy significantly prolongs the overall survival of patients with malignancy. Despite the progress in precise radiation delivery system, the healthy tissues and organs surrounding the tumor are inevitably subjected to radiation injury [2], among which RIV is very common. Currently, the pathogenesis of RIV is not fully elucidated. The primary mechanism may involve the pathological transformation of ECs, which are rather vulnerable to irradiation. Irradiation accelerates premature senescence and death of ECs via generation of excessive reactive oxidative species, increased DNA damage, and deregulation of age-related insulin/insulin-like growth factor-1 pathway [12, 13, 31]. The senescent ECs exhibit the impairment of cell-cell junctions, the increased expression of adhesion molecules, and the elevated release of inflammatory cytokines [13–15]. The alteration of EC biological function induces leukocyte trafficking, subendothelial lipid accumulation, and initiation of the atherogenic process. Moreover, EC death and detachment result in the exposure of thrombotic elements on the underlying basal membrane which promotes platelet activation and thrombosis [32]. Taken together, radiation-induced EC premature senescence, combined with EC death and exposure of thrombotic elements on the underlying basal membrane, leads to chronic inflammation and favors the development of vulnerable plaques. Therefore, promoting EC regeneration is an ideal approach to reduce the vascular radiotoxicity.

The role of MSCs in regenerative medicine has been well recognized [19, 20, 33]. MSCs are plastic adherent, nonhematopoietic cells that possess the capacity of self-renewal and multilineage differentiation. Although MSCs can secrete a plethora of bioactive factors to promote vascular regeneration, their differentiation to vascular cells plays a critical role as well [19, 20]. MSCs are demonstrated to differentiate into ECs and SMCs in vivo and in vitro. A series of studies have revealed that the endothelial differentiation of MSCs plays a critical role in vascular regeneration. MSCs are able to migrate to vein graft and acquire EC phenotype, which is associated with attenuated neointimal formation [34]. In vitro induction of endothelial differentiation before MSC transplantation greatly improves the efficacy of neointimal suppression [35]. The coculture with late-outgrowth ECs can induce endothelial differentiation of MSCs in vitro, and infusion of MSCs together with late-outgrowth ECs significantly inhibits SMC differentiation of MSCs and promotes their differentiation into ECs in vivo with the consequence of attenuated neointimal formation [36]. Of note, improvement of endothelial differentiation is also shown to strikingly alter the pro-atherogenic property of vascular resident stem cells. Adventitial progenitor cells often promote atherosclerosis by preferentially differentiating to neointimal SMCs. However, when adventitial progenitor cells are committed to endothelial fate by transfection with ETS variant 2, a member of E26 transformation-specific transcription factor family, they are no longer pro-atherogenic and even salutary to inhibit neointimal formation [37].

Recently, we genetically modified MSCs with HMGB1. HMGB1 is a small DNA-binding protein and can be released extracellularly to act as the damage-associated molecular pattern. The role of HMGB1 in tissue injury is dichotomous. On the one hand, HMGB1 contributes to proinflammatory response, which expands the possibilities for therapeutic modulation of HMGB1 activity to multiple inflammatory diseases [38–41]. On the other hand, HMGB1 promotes tissue repair by priming and recruiting stem cells [42]. We found that MSC-H cells showed a great potential for endothelial regeneration in a rat model of graft atherosclerosis [21]. MSC-H cells had a highly successful rate of cell implantation and EC differentiation, which was in accordance with significant inhibition of graft atherosclerosis in MSC-H-treated rats. Now we utilized MSC-H cells to treat RIV based on two reasons. First, if MSC-H cell therapy is only assessed in graft atherosclerosis, the efficacy of endothelial regeneration has to be interpreted in light of the disease-specific limitation. In other words, MSC-H cell therapy should be tested in other vasculopathy to verify its efficacy. Second, although RIV is a distinct entity, the injury of large arteries has the morphological patterns similar to atherosclerosis with other etiologies. For instance, both graft atherosclerosis and RIV are morphologically characterized by extensive inflammation, diffuse fibrosis, and neointimal formation. In the rat model, the irradiated aortas showed the gathering of abundant spindle-like cells and extracellular matrix mixed with some degree of inflammatory cell infiltration internal to the elastin membrane (Fig. 1a). The elastic fibers were decreased in the media and replaced by collagen fibers which eventually spread across the entire arterial wall. The injury of irradiated aortas presented herein was well-documented in humans [5]. It was very similar to graft atherosclerosis [21], although their etiological natures are quite different. Therefore, it was plausible that RIV benefits from MSC-H cell therapy as well. The current study turned out to support the hypothesis. After infusion of MSC-H cells versus MSC-C cells, the injured aortas showed a more significant relief with less intimal thickening and lower degree of vascular inflammation and fibrosis (Fig. 1a). Besides, the implanted cells were found to be at the greater quantity in MSC-H-treated group than MSC-C-treated group (Fig. 2a, b). MSC-H cells were preferentially recruited to the intima and differentiated to ECs (Fig. 2a, c), which were correlated with significant inhibition of neointimal formation in MSC-H-treated group (Fig. 1a). The findings agreed with the data obtained from graft atherosclerosis, suggesting MSC-H cell therapy potentially applicable to the vasculopathies with EC degeneration.

Given that the therapeutic efficacy of MSC-H cells was closely related to their potential of EC differentiation, we verified the differentiation capacity of MSC-H cells in vitro by using EC-MSC coculture system. The system has merit in that it captures the significant complexity of vascular microenvironment where MSCs grow as naturally in vivo. The CD31 expression of MSC-H cells was significantly increased at day 7 and reached the approximately three-fold higher level than that of MSC-C cells at day 14 (Fig. 2e). The endothelial differentiation was further validated by immunofluorescence for CD31 and LDL uptake assay (Fig. 2d, Additional file 2: Figure S2). Microarray analysis revealed that the number of differentially expressed genes in MSC-H cells was nearly two-fold higher than MSC-C cells after coculture with ECs. These genes were enriched in multiple biological processes and signaling pathways associated with stem cell development (Fig. 3a, b). On the one hand, the gene enrichment patterns shared some degree of similarity between MSC-H and MSC-C cells in that one-half of the most enriched GO terms and signaling pathways were consistent between them. However, among the shared enriched GO terms and signaling pathways, the significance gene counts were increased by the average of 1.7 and 1.4-fold, respectively, in MSC-H cells when compared to MSC-C cells. Therefore, HMGB1 modification was suggested to potentially augment MSC differentiation, which has recently been discovered to play a critical role in HMGB1 function. Despite the common knowledge of HMGB1 as an inflammatory mediator, a series of studies have supported the essential contribution of HMGB1 to tissue regeneration after damage [42–45]. HMGB1 forms a heterocomplex with SDF-1, binding to and signaling via CXCR4 to drive the differentiation of multiple stems and progenitor cells for the repair of injured tissues [42]. The mechanism is later known as the priming of stem cells in which HMGB1 transitions multiple quiescent stem cells from G_0_ to G_alert_ [42]. The primed G_alert_ cells enter the cell cycle more rapidly than quiescent stem cells, leading to accelerated tissue repair. Based on the theory, MSC-H cells were potentially subjected to the priming with HMGB1 modification, leading to their predisposition to endothelial differentiation upon angiogenic stimulation. On the other hand, the microarray analysis yielded a list of pathways that were exclusively activated in MSC-H cells and believed to have a close relationship with stem cell development (Fig. 3b). For instance, the MAPK signaling pathway was listed in the most enriched pathways of MSC-H cells rather than MSC cells. This pathway was previously shown to regulate osteoblastic differentiation and migration of MSCs upon HMGB1 stimulation [43, 44]. Another highly enriched pathway in MSC-H cells was p53 signaling which was crucial to the maintenance of stemness in embryonic stem cells and adult stem cells [46, 47]. The activation of p53 signaling leads to the rapid differentiation of stem cells [48]. By exploring the involvement of these pathways in the regulation of EC differentiation, we anticipate to have a clear knowledge of how MSC-H cells acquire the high potential of endothelial differentiation.

Another factor that contributed to the efficacy of MSC-H cells was their high motility, given that the MSC-H cells must migrate to vascular lesions prior to replacement of the injured ECs. We investigated whether the SDF-1/CXCR4 axis was involved in the process. The SDF/CXCR4 axis and HMGB1 are closely related in the regulation of stem cells. On the one hand, HMGB1 forms a heterocomplex with SDF-1 to bind to and signal via CXCR4, leading to activation of stem cells during tissue regeneration [42, 49]. On the other hand, HMGB1 promotes the production of SDF-1 by MSCs [50]. SDF-1 treatment stimulates CXCR4, which in turn generates endogenous SDF-1 [50]. This positive feedback mechanism may render CXCR4 very sensitive to HMGB1 stimulation and upregulated as well. In our study, MSC-H cells really showed a higher level of CXCR4 than MSC-C cells (Fig. 4c). Moreover, the expression of SDF-1 was significantly increased in the irradiated aortas (Fig. 4a, b). These findings suggested that local migration of MSC-H cells was enhanced possibly via regulation of the SDF-1/CXCR4 axis. To better support the assumption, we performed the SDF-1-induced transwell migration assay. Both MSC-H cells and MSC-C cells migrated with SDF-1 stimulation in a dose-dependent manner, although the response of MSC-H cells was much stronger than MSC-C cells (Fig. 4c). After the SDF-1/CXCR4 axis was blocked by AMD3100, the migration of MSC-H cells was strikingly inhibited (Fig. 4d). Therefore, the high mobility of MSC-H cells was at least partly regulated by SDF1/CXCR4 axis. It might be argued that MSC-H cell migration was attributed to activation of CXCR7, another SDF-1 receptor. To rule out the possibility, we measured the expression of CXCR7 on MSC-H and MSC-C cells and found that they both expressed a very low level of CXCR7 (Fig. 4c). Besides, the expression of CXCR7 was still not altered when endothelial differentiation was stimulated by EC-MSC coculture (Fig. 4c). These findings suggested that SDF-1/CXCR7 axis was not likely to contribute to the high motility of MSC-H cells.

Despite the aforementioned benefits, MSC-H cell infusion was supposed to generate more favorable effects on RIV. Not all MSC-H cells homed and differentiated to ECs if not entirely attributable to cell death. So how did the rest of MSC-H cells work? The irradiated aortas showed extensive infiltration of MPO-positive cells and high expression of proinflammatory cytokines like TNF-α, TGF-β, and IL-1β (Fig. 1a, c). On the one hand, it suggested oxidative stress and inflammatory response following irradiation. On the other hand, this to some extent reflected the accumulation of senescent cells in irradiated aortas, leading to radiation-associated functional decline, given that the senescent cells are known to accumulate in vivo after irradiation and capable to increase the production of reactive oxygen species and proinflammatory cytokines [51]. Indeed, the implanted MSCs may differentiate to new vascular cells in vivo, and by that way, they compensate to some degree for loss of vascular cells which are radiosensitive and subjected to senescence and cell death following irradiation. But the circulating MSCs are also believed to take effect via secreting bioactive factors that are immunomodulatory and trophic (regenerative) [19]. In the scenario, the therapeutic efficacy of MSC-H cells might be mediated by a paracrine mechanism through which an angiogenic microenvironment was created to stimulate endogenous vascular progenitors.

Finally, we investigated whether MSC-H cells were activated by a HMGB1 autocrine loop. Our recent work found that addition of HMGB1 protein to the MSC culture generated the physiological effects similar to the genetic modification of MSCs with HMGB1 gene [21]. Blocking HMGB1 receptor RAGE inhibited the response of MSCs to the stimulation of HMGB1 protein. Therefore, we proposed that MSC-H cells might be regulated by HMGB1 autocrine signaling, in which a great deal of extracellular HMGB1 produced by MSC-H cells in turn activated MSC-H cells, leading to more HMGB1 production and release via a positive feedback loop. The current study supported our assumption. The level of cytosolic HMGB1 was four-fold higher in MSC-H cells than MSC-C cells, but HMGB1 levels in the nucleus were not different between them. The production of extracellular HMGB1 by MSC-H cells was six-fold higher than MSC-C cells. Moreover, when extracellular HMGB1 was blocked by HMGB1 Ab, the migration and endothelial differentiation of MSC-H cells were strikingly inhibited. Collectively, these findings suggested that HMGB1 autocrine signaling existed in MSC-H cells and contributed to their high mobility and endothelial differentiation.

## Conclusion

**Fig. 6:**
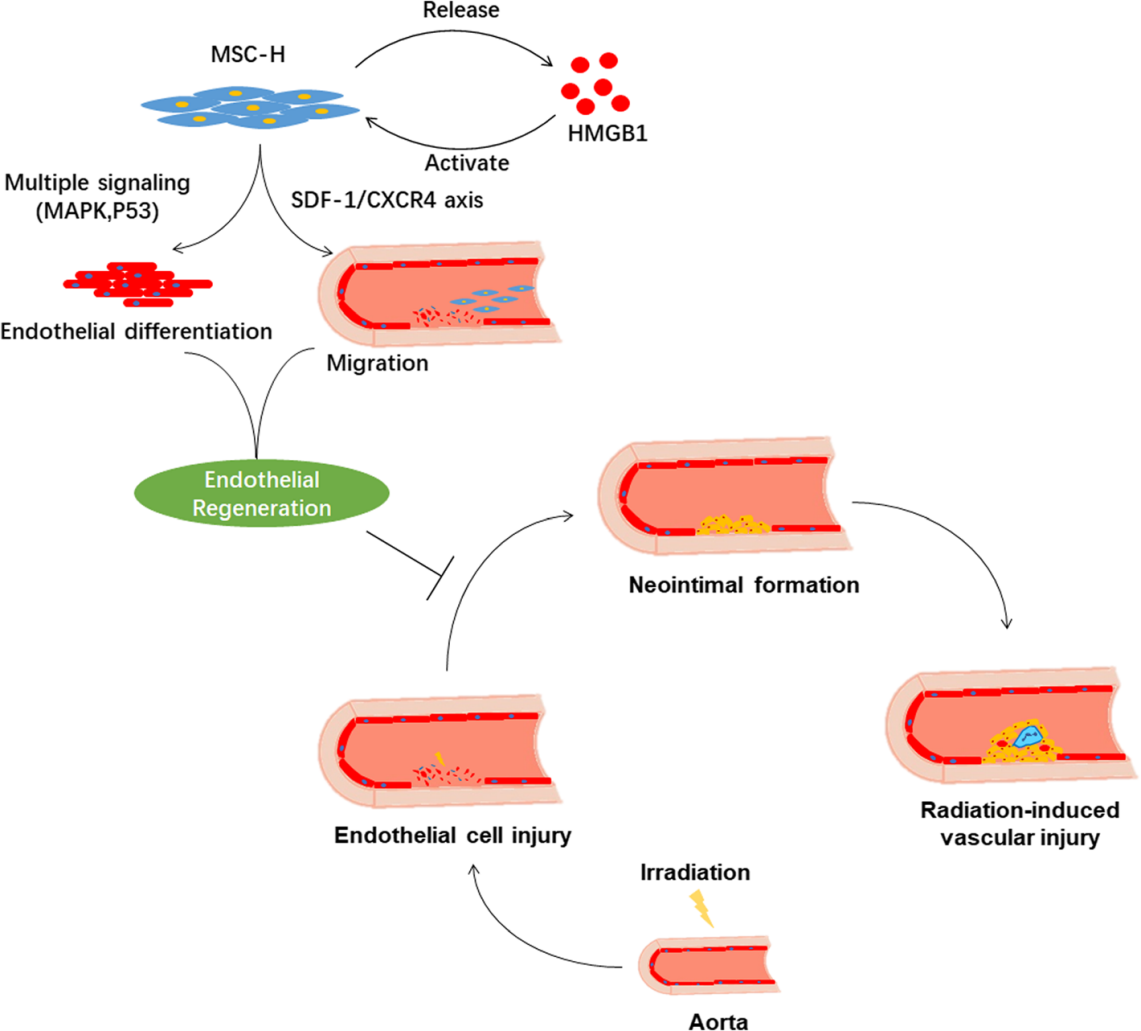
Illustration of possible mechanisms that HMGB1 modification improves the efficacy of mesenchymal stem cells (MSCs) in treating the radiation-induced vascular injury. HMGB1-modified MSCs (MSC-H cells) produce and actively release HMGB1 to the extracellular milieu. Extracellular HMGB1 binds to and signals via its receptors on MSC-H cells which are activated to generate more HMGB1 via a positive feedback loop. Besides, extracellular HMGB1 primes MSCs to increase their mobility and differentiation toward endothelial lineage. Multiple pathways are involved in the process, including the SDF-1/CXCR4 axis, MAPK, and p53 signaling. Thus, the ability of MSCs to regenerate is improved and later translated to their high efficacy in inhibiting neointima formation after irradiation

We tested the efficacy of MSC-H cell therapy in RIV by using a rat model. Infusion of MSC-H cells significantly attenuated neointimal formation, which was associated with their high motility and endothelial differentiation potential. Multiple pathways that contributed to the bioactivity of MSC-H cells were suggested, such as MAPK and p53 signaling, SDF-1/CXCR4 axis, and HMGB1 autocrine signaling (Fig. 6). The possible paracrine effect of MSC-H cells on vascular resident progenitors deserved further investigation in the context of their efficacy on vascular regeneration.

## Additional files

Additional file 1: **Table S1.** Animal groups and treatment protocols. (DOCX 15 kb)

Additional file 2: **Figure S1.** The sections of the aortas from RT, Sham RT, Sham RT + MSC-C, and Sham RT + MSC-H groups were examined by fluorescent microscopy. They served as negative control for RT + MSC-C and RT + MSC-H groups. Sham RT + MSC-C and Sham RT + MSC-H groups consisted of the rats which were infused MSCs at the same dosages as RT + MSC-C and RT + MSC-H groups, respectively, after Sham irradiation. The fluorescent microscopy was adjusted to view GFP and DAPI labels. There were no GFP-labeled cells in the sections. The images were representative of the examination of eight rats for each group. (PDF 1499 kb)

Additional file 3: **Figure S2.** The cell uptake of DiI-Ac-LDL was examined at day 14 after EC-MSC coculture. (A) MSC-H cells had a significantly higher rate of DiI-Ac-LDL uptake than MSC-C cells. (B) The population of cells with DiI-Ac-LDL uptake was greatly reduced after MSC-H cells were treated HMGB1 Ab. HMGB1 Ab treatment hardly affected endothelial differentiation of MSC-C cells. The images were representative of three experiments for each group. (PDF 778 kb)

Additional file 4: Data package includes the dataset of microarray analysis. The differentially expressed genes were listed together with heatmaps. (RAR 12339 kb)

## Abbreviations

CXCR4: CXC chemokine receptor 4
CXCR7: CXC chemokine receptor 7
DAPI: 4′,6-Diamidino-2-phenylindole
DMEM: Dulbecco’s modified Eagle’s medium
EC: Endothelial cell
GAPDH: Glyceraldehyde-3-phosphate dehydrogenase
GFP: Green fluorescent protein
GO: Gene Ontology
HMGB1: High mobility group box 1
HMGB1 Ab: HMGB1 antibody
IL-1β: Interleukin 1β
KEGG: Kyoto Encyclopedia of Genes and Genomes
LDH: Lactate dehydrogenase
LDL: Low-density lipoprotein
MPO: Myeloperoxidase
MSC: Mesenchymal stem cells
MSC-C: Mesenchymal stem cells transfected with vehicle control vectors
MSC-H: HMGB1 modified mesenchymal stem cells
RIV: Radiation-induced vascular injury
SDF-1: Stromal-derived factor 1
Sry: Sex determination region on the Y chromosome
TGF-β: Transforming growth factor β
TNF-α: Tumor necrosis factor α
