# Supplementary material for: HMGB1-modified mesenchymal stem cells attenuate radiation-induced vascular injury possibly via their high motility and facilitation of endothelial differentiation
Source: Stem Cell Res Ther. 2019 Mar 13;10:92. doi: 10.1186/s13287-019-1197-x (PMC6416980; doi:10.1186/s13287-019-1197-x)
Supplement: Supplementary file 1 — Table S1. Animal groups and treatment protocols. (DOCX 15 kb) [file 13287_2019_1197_MOESM1_ESM.docx]

Table S1: Animal groups and treatment protocols

| Group | n | Irradiation | MSC infusion |
| --- | --- | --- | --- |
| Sham RT | 8 | - | - |
| RT | 8 | + | - |
| RT+MSC-C | 8 | + | 2 × 10^6^ MSC-C cells per dose × 4 doses |
| RT+MSC-H | 8 | + | 2 × 10^6^ MSC-H cells per dose × 4 doses |

Note: The symbols of ‘+’ and ‘-’ represented that the referred treatment was given or not, respectively.
