# Supplementary figures and images for: HMGB1-modified mesenchymal stem cells attenuate radiation-induced vascular injury possibly via their high motility and facilitation of endothelial differentiation
Source: Stem Cell Res Ther. 2019 Mar 13;10:92. doi: 10.1186/s13287-019-1197-x (PMC6416980; doi:10.1186/s13287-019-1197-x)

**GFP****DAPI****Merge****Sham RT**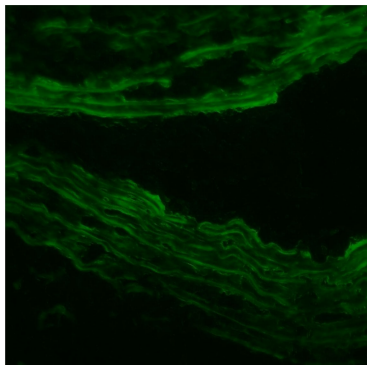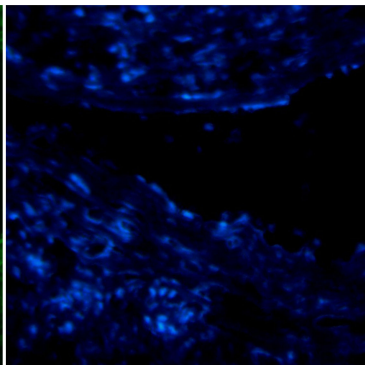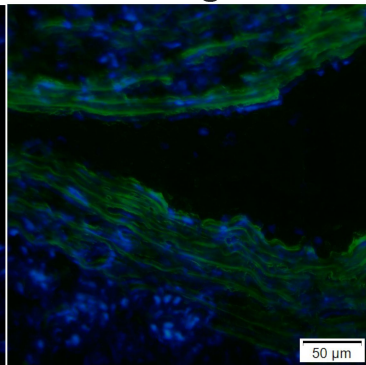**RT**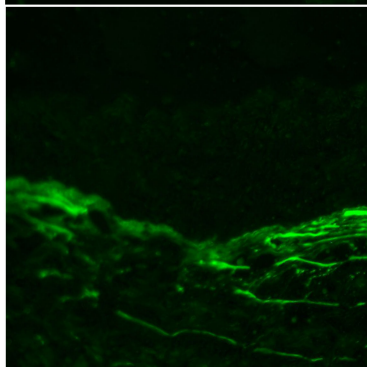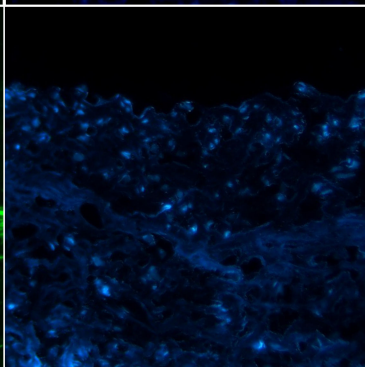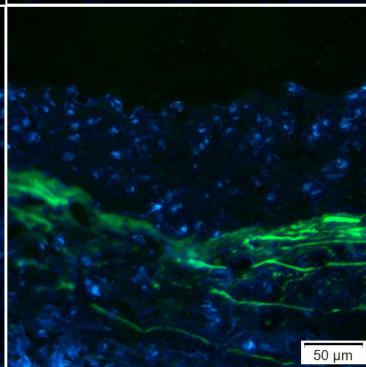**Sham RT + MSC-C**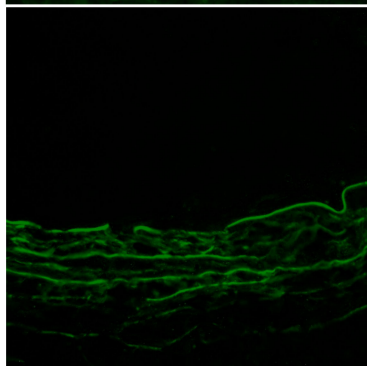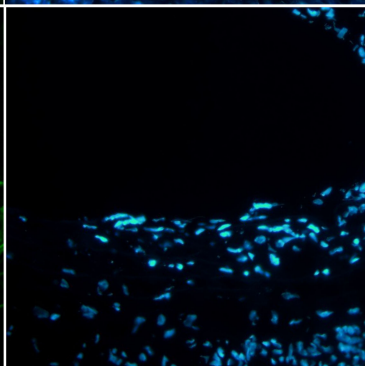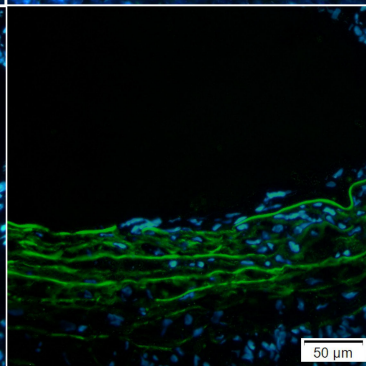**Sham RT + MSC-H**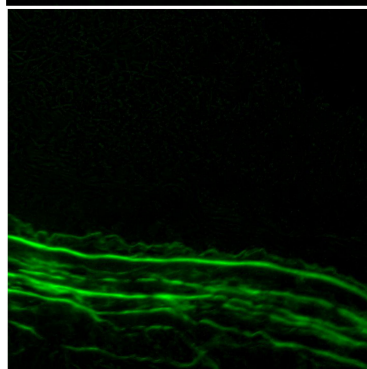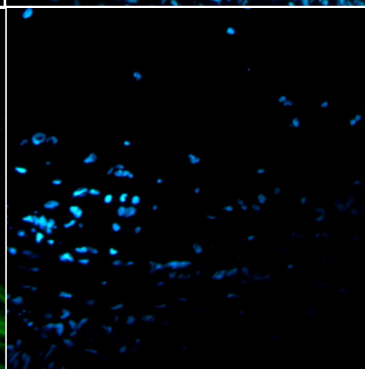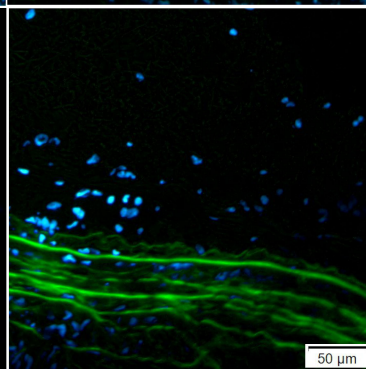

Supplement: Supplementary file 2 — Figure S1. The sections of the aortas from RT, Sham RT, Sham RT + MSC-C, and Sham RT + MSC-H groups were examined by fluorescent microscopy. They served as negative control for RT + MSC-C and RT + MSC-H groups. Sham RT + MSC-C and Sham RT + MSC-H groups consisted of the rats which were infused MSCs at the same dosages as RT + MSC-C and RT + MSC-H groups, respectively, after Sham irradiation. The fluorescent microscopy was adjusted to view GFP and DAPI labels. There were no GFP-labeled cells in the sections. The images were representative of the examination of eight rats for each group. (PDF 1499 kb) [file 13287_2019_1197_MOESM2_ESM.pdf]

**A****MSC-C**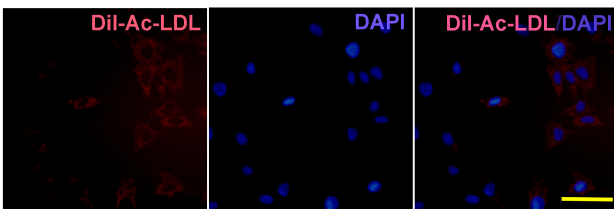**MSC-H**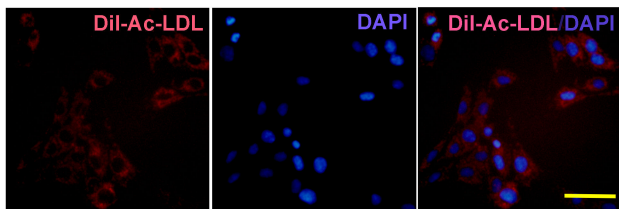**B****MSC-C + HMGB1 Ab**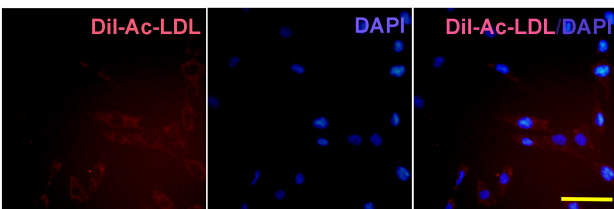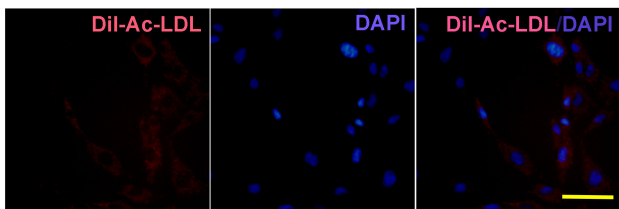**MSC-H**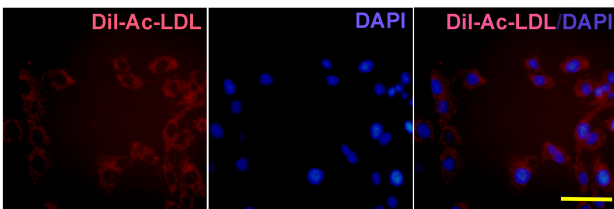**MSC-H + HMGB1 Ab**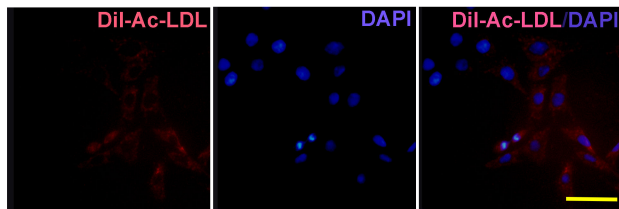

Supplement: Supplementary file 3 — Figure S2. The cell uptake of DiI-Ac-LDL was examined at day 14 after EC-MSC coculture. (A) MSC-H cells had a significantly higher rate of DiI-Ac-LDL uptake than MSC-C cells. (B) The population of cells with DiI-Ac-LDL uptake was greatly reduced after MSC-H cells were treated HMGB1 Ab. HMGB1 Ab treatment hardly affected endothelial differentiation of MSC-C cells. The images were representative of three experiments for each group. (PDF 778 kb) [file 13287_2019_1197_MOESM3_ESM.pdf]
